# Utilization of Stainless-steel Furnace Dust as an Admixture for Synthesis of Cement-based Electromagnetic Interference Shielding Composites

**DOI:** 10.1038/s41598-017-15779-7

**Published:** 2017-11-13

**Authors:** Yong Fan, Ling Zhang, Vladimir Volski, Guy A. E. Vandenbosch, Bart Blanpain, Muxing Guo

**Affiliations:** 1 Department of Materials Engineering, KU Leuven, Kasteelpark Arenberg 44 us 2450, B-3001 Heverlee, Belgium; 2 Institute of Multidisciplinary Research for Advanced Materials, Tohoku University, 2-1-1 Katahira, Aobaku, Sendai, 980-8577 Japan; 3 Department of Electrical Engineering, KU Leuven, B-3001 Heverlee, Leuven Belgium

## Abstract

Electromagnetic interference (EMI) shielding receives attention due to the increasing abundance of electronics. The Cement based material can obtain EMI shielding properties through the use of appropriate “fillers” such as carbon, metal, and ferrite. As the most important by-product of stainless steelmaking operations, through the metal droplets and ferrite that it contains, stainless-steel dust can be considered as a potential filler for EMI shielding applications. We have therefore utilized stainless-steel dust as an admixture for the synthesis of cement-based EMI shielding composites and show that it raises the EMI shielding effectiveness. In particular, a 45 mass pct of stainless-steel dust mixture of 5 mm thickness results in the enhancement of EMI shielding effectiveness to 6–9 dB as tested in the frequency range of 500 MHz–1.5 GHz.

## Introduction

**Figure 1:**
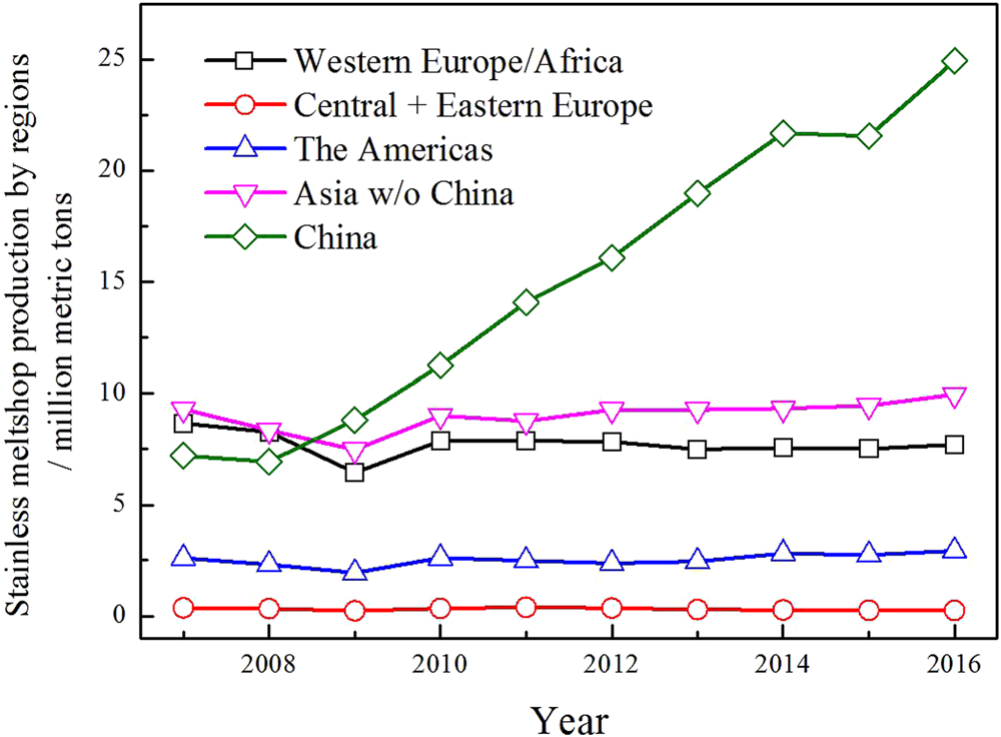
The stainless steel melt-shop production by regions.

Nowadays, the production of stainless steel is one of the most important and fastest growing branches of the metallurgical industry all over the world. Figure 1 illustrates the stainless steel meltshop production by regions. Worldwide stainless steel production reached approximately 45.8 million metric tons in 2016, up from the 41.5 million metric tons produced in 2015. The rise was attributed to an increase in output in China - the world’s largest stainless steel producing country - where production rose by over 10 pct year-on-year to approximately 24.9 million metric tons in 2016 ^1^. In the stainless steel making process, between 30 and 70 kg of dust or fine waste is generated per ton of steel. Stainless steel dust came from the flue gas because of the violent agitation of molten steel in the furnace, was the mixtures of metals, slags, and gangues. It newly formed, in the form of fine particulates, being carried away from the furnace loads following with exhaust gases in the pipeline, and finally collected by bag filters or electrostatic precipitators^2^. In general, the exact composition of stainless-steel dust is widely variable, and quite complex depending on the scrap types and other process input materials^3^.

In view of developing a sustainable steel production, the potential of its residues as a secondary resource must be considered to avoid landfilling. Recycling of stainless dust, was studied by many researchers^4^, makes it possible to recover valuable metals such as iron, chromium, nickel, and zinc, whose are needed in stainless steel manufacture and could be returned to the production procedure. This article provides a proof-of-principle of a new high-value added application for stainless-steel dust, namely, the use of stainless-steel dust as an admixture for enhancing electromagnetic interference (EMI) shielding.

EMI shielding receives mounting attention due to the increasing presence of electronics, such as communication and transportation systems, wireless LAN networks, and personal digital devices. in both military application and civil life^5^. Accordingly, the EMI shielding material plays a paramount role in electromagnetic pollution controlling, communication security, and ground/underground substation containing central processing electronics, which are crucial to power transmission and telecommunication. Many researchers^6^ have investigated cement based material which is not only a structural material, but also, have EMI shielding properties through introducing “fillers”, for instance, carbon^7^, metal^8^, and ferrite^9^ materials.

In general, the primary mechanism of EMI shielding is based on reflection of the radiation through mobile charge carriers such as electrons, which interact with the electromagnetic field^10^. There is no doubt that high conductivity materials such as carbon or metal are suitable as fillers^11^. The second mechanism of EMI shielding relies on absorption of the radiation through electric and/or magnetic inlets that interact with the electromagnetic fields. The magnetic dipoles might be provided by hematite, magnetite or other ferrites with high magnetic permeability^12^. Cao and Chung^13^ have found that iron oxide in fly ash can enhance shielding through radiation absorption. The effectiveness of fly ash for shielding is low compared to that of conductive admixtures such as carbon based fillers^14^. Nevertheless, fly ash is much lower in cost than the conductive admixtures.

Stainless-steel dust is generated during the violent agitation of molten steel in the stainless steel making process and is collected by bag filters or electrostatic precipitators. This dust contains both metal droplets and ferrites that could be a potential filler for EMI shielding function. Stainless-steel dust can be mixed into the cement matrix for obtaining EMI shielding effect, which provides a practicable and value-added way for its reutilization and/or recycling. In this paper, the effectiveness of stainless-steel dust as an admixture for the synthesis of cement-based EMI shielding composites is investigated experimentally.

## Experimental Method

The stainless-steel dust obtained from a stainless steelmaker in Belgium was used in the present experiments. The chemical composition of the dust was determined by inductively coupled plasma optical emission spectroscopy (ICP-OES, Varian 720 ES, USA), was illustrated in Table 1. The mineral composition was determined with the use of X-ray diffraction (XRD, 40 kV, 30 mA, Cu-Kα, SEIFERT 3003 T/T, Germany). The morphology and mineral phase analysis of the dust particulates were studied by scanning electron microscope and energy dispersive spectrometer (SEM-EDS, XL30FEG, Philips, USA) and electron probe microanalysis (EPMA, JXA-8530F, Jeol, Japan).

**Table 1:** The chemical composition of the stainless steel dust (mass pct).

TFe	Cr	MgO	CaO	SiO_2_	ZnO
33.5	9.6	8.4	20.6	4.3	5.1

A typical Portland cement was employed in the experiments. 15, 30 and 45 mass pct of stainless-steel dust were respectively added into the cement and mixed. 200 g each of the mixtures was used to prepare a specimen of 5 mm thickness with a final ±0.3 mm in precision. After mixing and homogenizing the cement and stainless-steel dust, water was added to achieve a moisture content of approximately 30 mass pct. Shaping was performed with a Teflon mold with dimensions of 140 mm in side length. Then, specimens were dried for 48 hours in the fuming cupboard, demolded, and cured finally at room temperature and humidity. Magnetite (Fe_3_O_4_, >99.5 pct, Sigma-Aldrich, USA), hematite (Fe_2_O_3_, >99 pct, Sigma-Aldrich, USA), and metallic iron (Fe, >99.5 pct, Emsure, Germany) were respectively used as filler for the sake of comparison in proportions by weight relative to cement of 15 mass pct. The morphology and mineral phase analysis of a typical sample obtained by the different conditions were characterized using SEM-EDS and EPMA.

**Figure 2:**
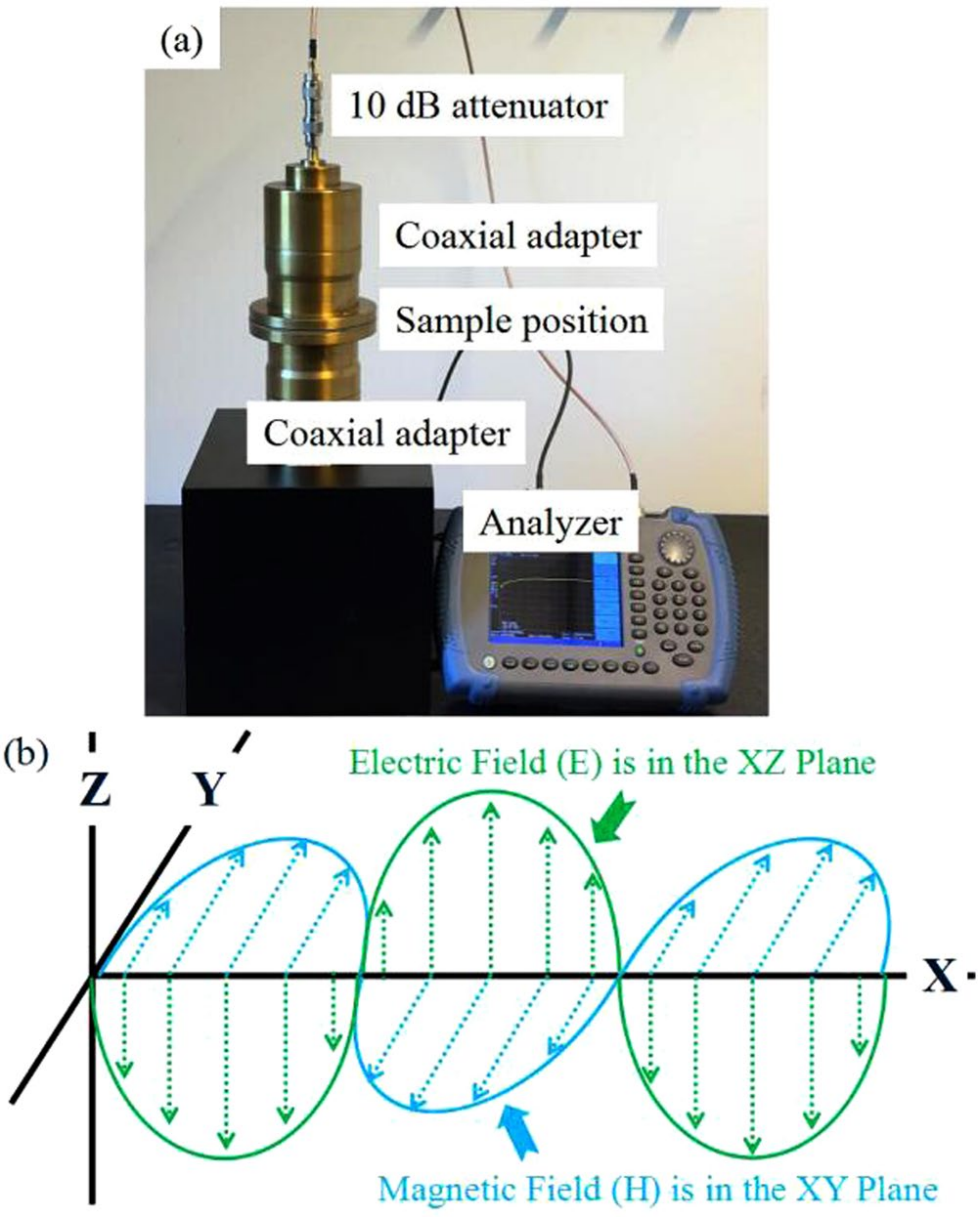
The coaxial adapters for EMI shielding measurement (**a**) and its electric (E) and magnetic (H) field distribution (**b**).

The shielding effectiveness was measured using a set-up based on the shielding effectiveness test fixture EM-2017A as shown in Fig. 2(a). This set-up is composed of two coaxial adapters, two attenuators and a spectrum analyzer (Keysight N9344C) with a built-in tracking generator. The 10 dB, 50 Ω attenuator is used on each end of the adapters. Their main purpose in this system is for impedance matching and used to isolate the specimen holder from the signal generator and the receiver, in case of a large reflection of energy back into the signal generator when testing a material. The distribution of the electric field (E) and magnetic field (H) in the coaxial adapters is shown in Fig. 2(b) ^15^. The shielding effectiveness of the specimens was determined by two consecutive transmission measurements between the two adapters with and without a sample present. The shielding effectiveness of the sample is calculated based on the ratio between the two measured received power levels.

## Results and Discussion

### The stainless-steel dust particulates

The compositional analysis confirms the presence of iron, chromium, calcium, magnesium, silicon, and zinc as the major metallic element in the stainless-steel dust, and numerous other elements in lower concentrations, such as copper, manganese, lead, aluminum, and nickel. The presence of heavy metals can pose threats to the environment and human health, therefore this waste is considered hazardous in many countries. The reusing and recycling of this waste should be carefully handled and studied. Although the environmental impacts are beyond the scope of the present study, future attention would have to be paid to these issues.

**Figure 3:**
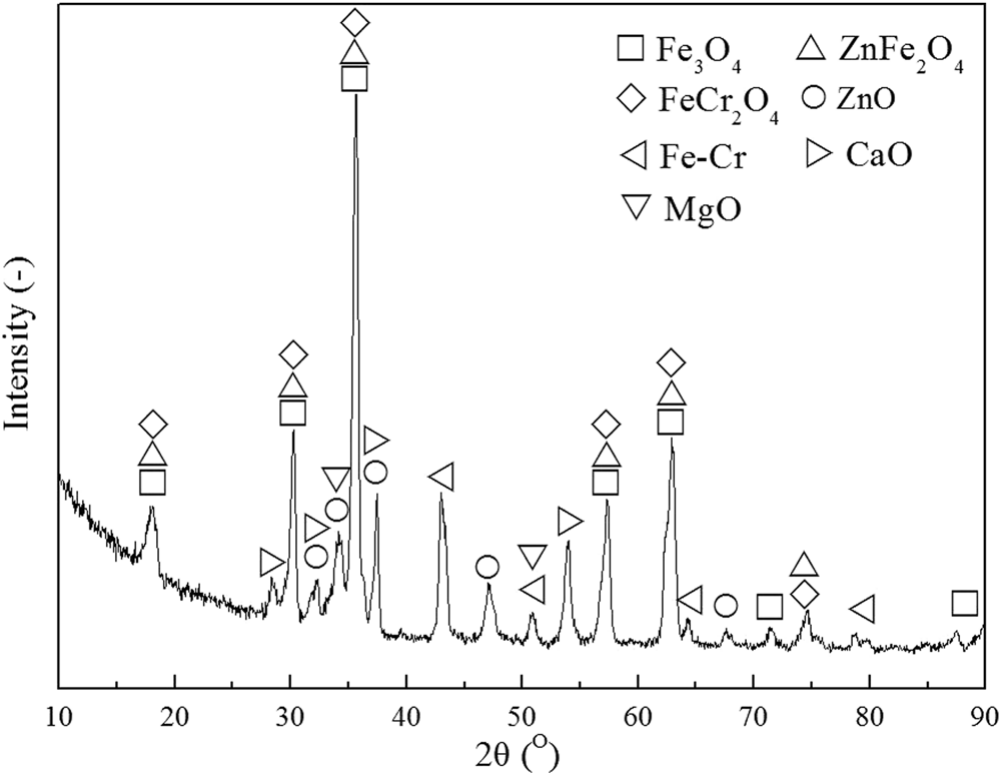
XRD patterns of the stainless-steel dust.

As was detected with XRD and shown in Fig. 3, major components of the stainless-steel dust are solid iron oxides, but also chromium and zinc containing iron oxides are present. Fe is mostly found as magnetite (Fe_3_O_4_), chromite (FeCr_2_O_4_), franklinite (ZnFe_2_O_4_) and Fe-Cr Alloy. There were also certain amounts of CaO and MgO present.

**Figure 4:**
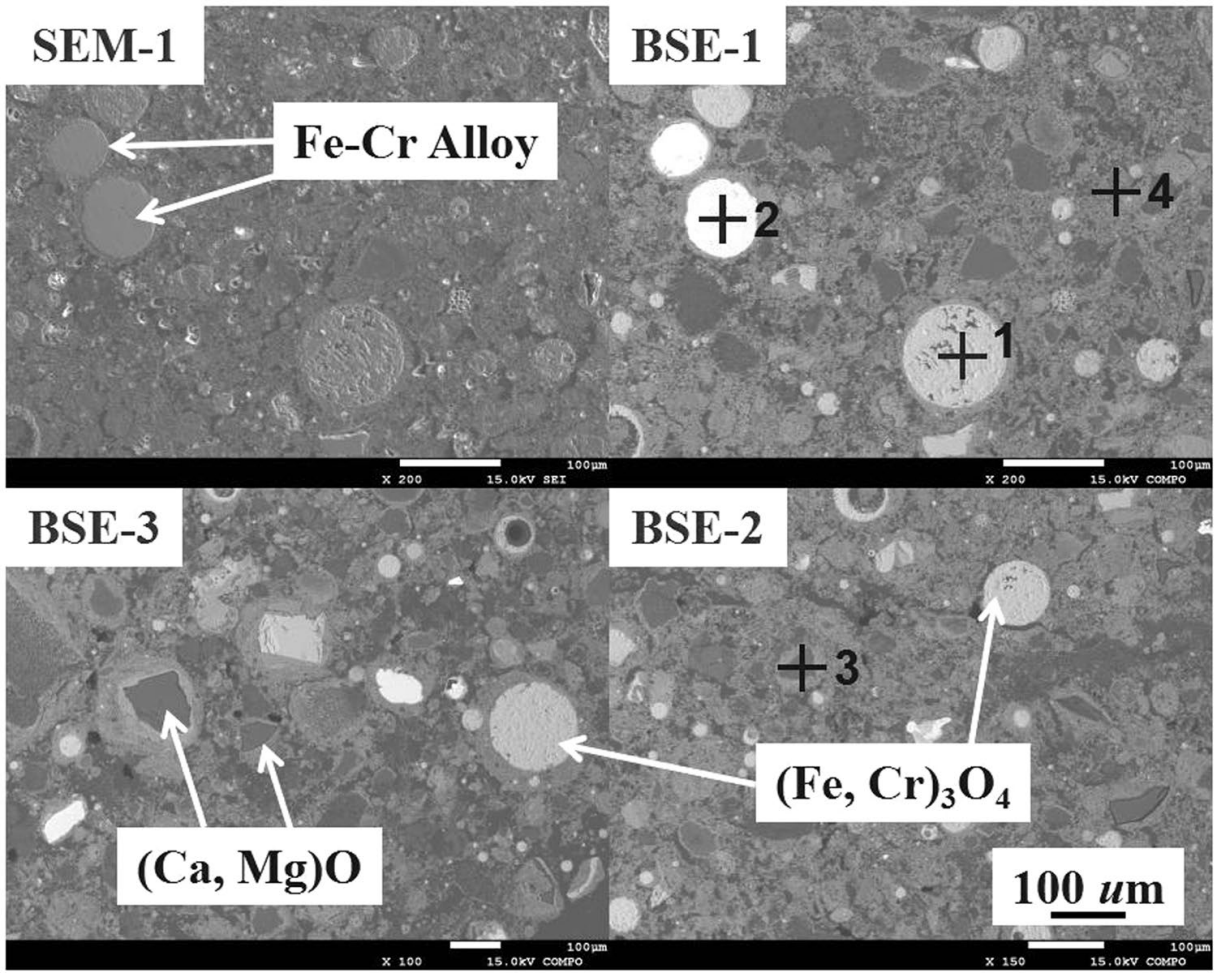
SEM and BSE images of stainless-steel dust particulates.

Typical SEM and BSE images and point analysis of stainless-steel dust particulates are shown in Fig. 4 and Table 2, which confirm the results from XRD. There are found four major characteristic crystal phases in this typical stainless-steel dust sample, i.e. (1) a round shape magnetite and/or chromite crystal (Fig. 4, phase 1) with less than 100 *u*m diameter and a rough surface; (2) metallic Fe-Cr alloy round particles (Fig. 4, phase 2) with a smooth surface; (3) lime and magnesia crystals with an irregular square shape (Fig. 4, darker phase 3) have a rather wide size distribution with an average of approximately 100 *µ*m; (4) Another phase (Fig. 4, phase 4) has on average a small crystal size and usually clusters together. It has a complicated composition with Fe as the main metallic element but also containing amongst others Cr and Zn etc., briefly whiten as (Fe, Cr, Zn)_3_O_4_. Due to the complex morphology of stainless-steel dust, other trace element phases are present which we will not further discuss here.

**Table 2:** Point analysis of typical phases of stainless-steel dust particulates in Fig. 4 (mass pct).

	Fe	Cr	Mg	Ca	Si	Zn
1	63.9	11.9	—	—	—	—
2	84.9	15.1	—	—	—	—
3	—	—	38.4	27.7	—	—
4	46.4	14.4	1.5	1.2	0.7	14.8

### Morphology of the Cement-based composites

**Figure 5:**
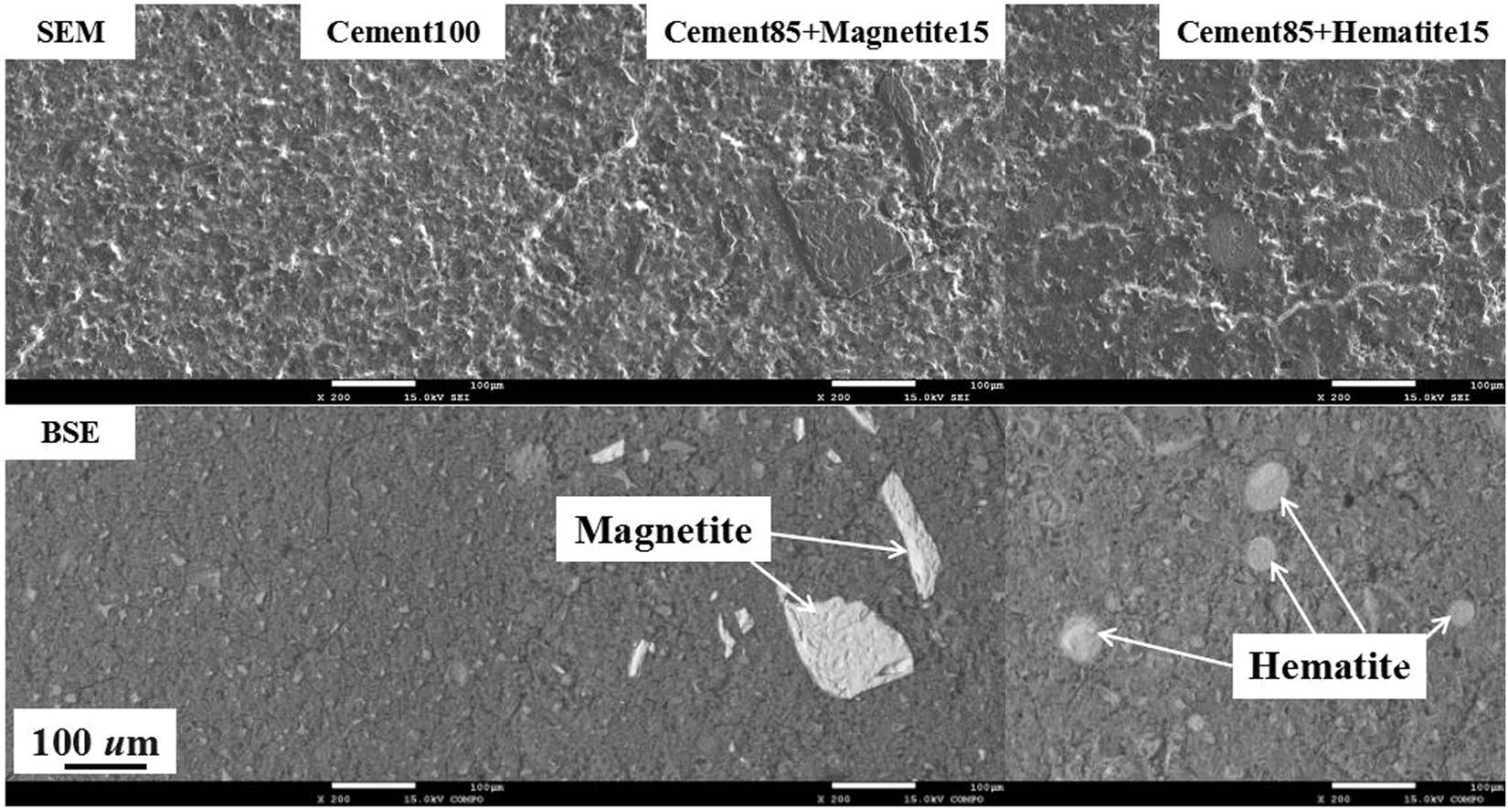
SEM and BSE images of reference samples.

Figure 5 shows the morphology of reference samples, namely, pure cement (Cement100), 15 mass pct magnetite (Cement85 + Magnetite15) and hematite (Cement85 + Hematite15) cement-based composites. The chemical composition of the pure cement was as follows: CaO = 45.8 mass pct, SiO_2_ = 38.4 mass pct, Al_2_O_3_ = 9.4 mass pct, and Fe_2_O_3_ = 6.4 mass pct. These samples were measured in a series with dust filler sample for comparison.

**Figure 6:**
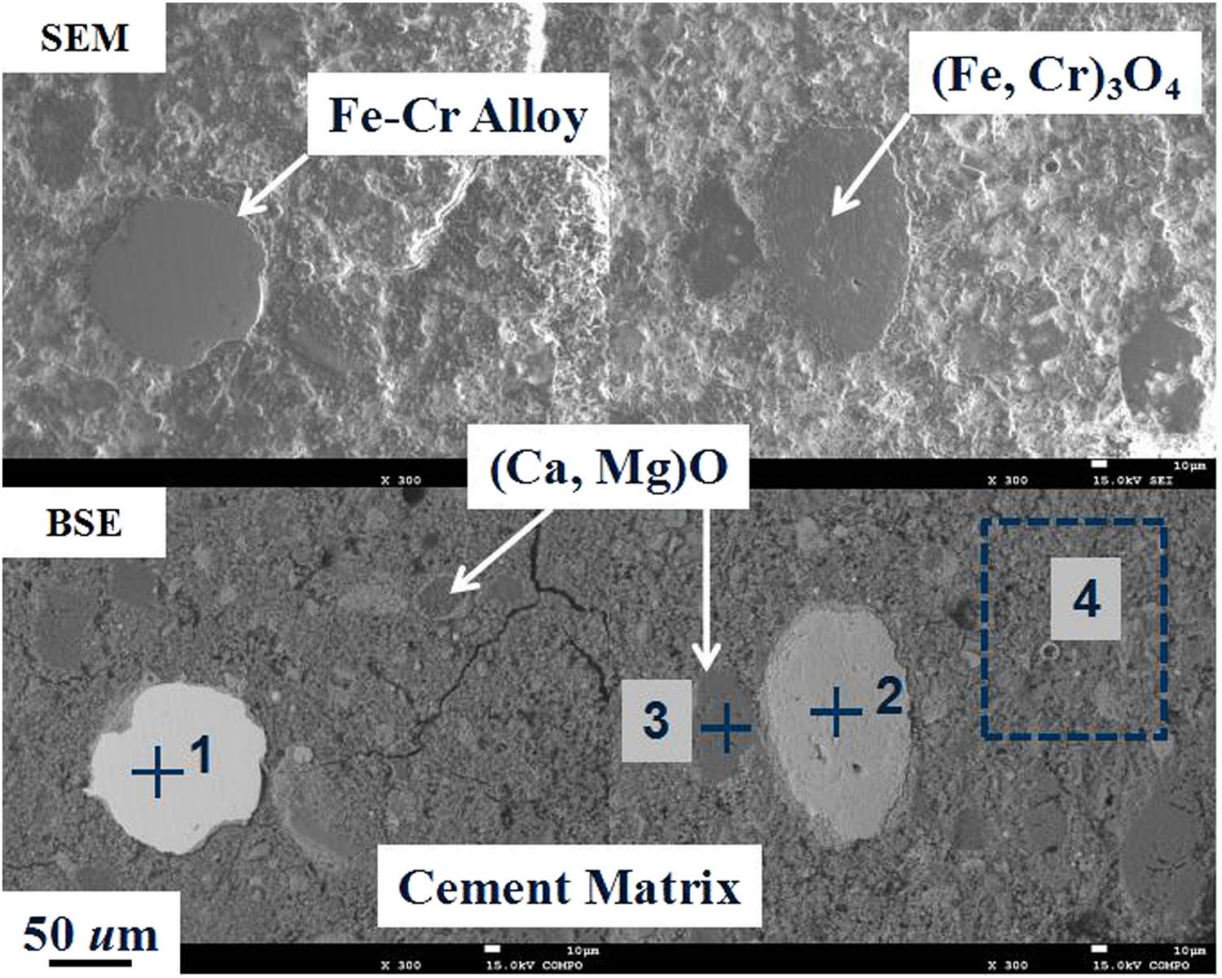
SEM and BSE images of the sample with 45 mass pct of stainless-steel dust.

Figure 6 and Table 3 shows the morphology and point analysis of some phases in the sample with 45 mass pct of stainless-steel dust mixed with pure cement (Dust 45). Characteristic particulates from the dust could be found in this sample, namely, Fe-Cr Alloy (Fig. 6, phase 1), (Fe, Cr)_3_O_4_ (Fig. 6, phase 2), and (Ca, Mg)O (Fig. 6, phase 3). The chemical composition of cement matrix (Fig. 6, area 4) had some variations due to the mix with stainless-steel dust.

**Table 3:** Point analysis of typical phases of sample in Fig. 6 (mass pct).

	Fe	Cr	Mg	Ca	Si	Zn	Al
1	85.4	13.6	—	—	—	—	—
2	31.1	38.8	—	1.0	—	—	—
3	5.2	0.9	24.8	20.8	2.3	1.3	2.3
4	12.0	3.1	1.9	29.2	6.2	2.2	1.8

### EMI shielding effectiveness and mechanism

**Figure 7:**
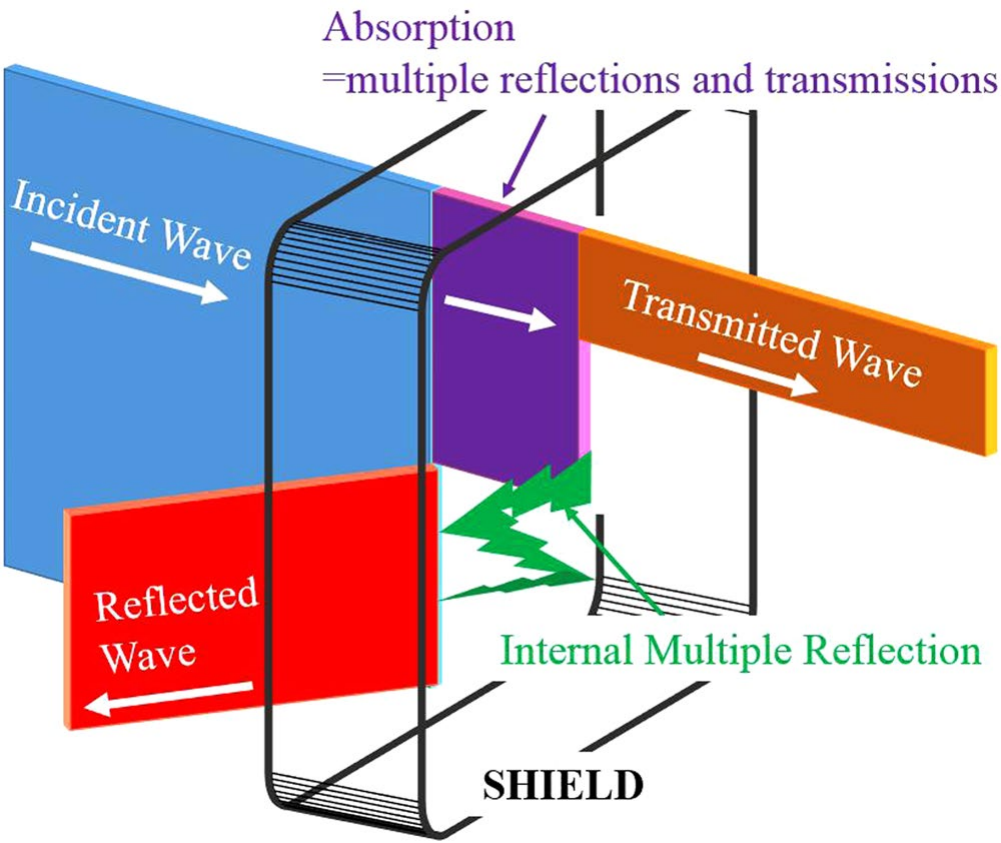
Attenuation of an electromagnetic wave by a shield.

The EMI of a shielding material is related to the residual traveling energy after applying the shield. The residual energy is the energy that is neither reflected nor absorbed by the shield but that emerges out of the shielding material. The attenuation of an electromagnetic wave occurs by three mechanisms, as shown in Fig. 7, which are reflection loss, absorption loss, and internal multiple reflections. The reflection loss is caused by the reflection at the surface of the shield. The absorption loss of the waves as it proceeds through the shield is the additional effects of multiple reflections and transmissions in the interior of the shield. EMI shielding effectiveness (SE) of a material is measured in decibels (dB), defined as a ratio of the transmitted to the incident power, and given as:

1 $$SE=-20\,\mathrm{lg}({P}_{a}/{P}_{b})$$

Where *P*_*a*_ and *P*_*b*_ are the transmitted and incident electromagnetic power, respectively. Total SE is a summation of shielding by absorption, reflection and multiple reflections/transmissions in the interior of the material.

Coaxial transmission line measurement in the present study is performed in the frequency range of 500 MHz–1.5 GHz. These frequency ranges are considerably important for commercial applications, such as TV signal, Mobile phone, wireless LAN and radar image.

**Figure 8:**
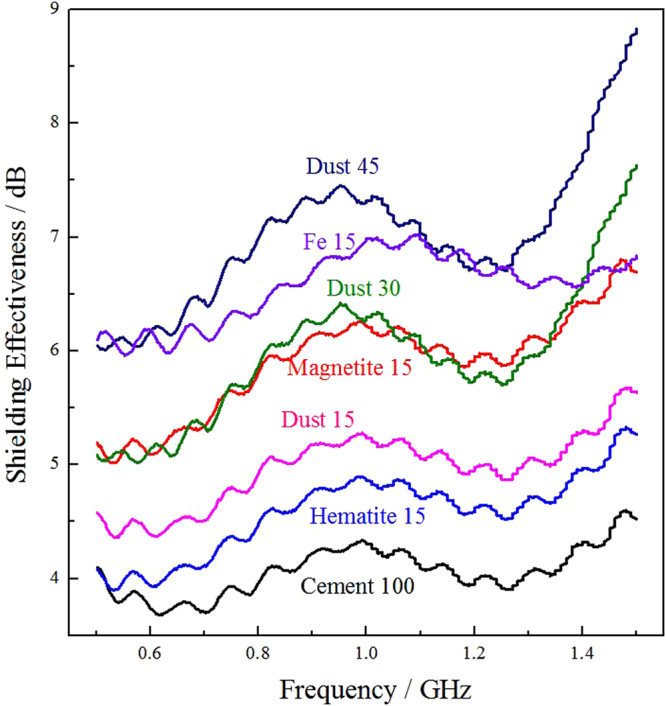
The shielding effectiveness of reference sample and dust filler sample of 5 mm thickness.

The impact on the electromagnetic shielding of the reference sample and dust filler sample of 5 mm thickness was analyzed as shown in Fig. 8. In general, the shielding effectiveness of a cement based material correlates closely to the electric conductivity and the electromagnetic parameters of the composite. The cement matrix (Fig. 8, Cement100) is only slightly conductive, leading to its relatively low shielding effectiveness. Ferrite like magnetite and hematite is distinguished magnetic loss absorbent, which would result in electromagnetic energy absorption by polarization mechanisms such as hysteresis loss and magnetic domain resonance. Magnetite has stronger magnetism than hematite and shows an enhanced shielding effectiveness when it added (Fig. 8, Magnetite15 & Hematite15) as a filler into the cement composite. Metal materials belong to dielectric loss absorbents, which mainly attenuate electromagnetic energy by electronic and ionic polarization. Metallic iron filler (Fig. 8, Fe 15) illustrated a promising electromagnetic shielding effectiveness than ferrite fillers. The dust filler with different weight mass percentage into the cement, namely 15 mass pct (Fig. 8, Dust15), 30 mass pct (Fig. 8, Dust30), and 45 mass pct (Fig. 8, Dust45) indicated enhancement of the shielding effectiveness to the cement matrix. The addition of stainless-steel dust alone to a cement composite sample raises the shielding value. In particular, a 45 mass pct of stainless-steel dust mixture results in the enhancement of EMI shielding effectiveness to approximately 6–9 dB as tested in the frequency range of 500 MHz–1.5 GHz, emphasizes the incident electromagnetic wave has been reduced by approximately 60 pct. This is believed to be attributed to the metal droplets and ferrite embedded in the sample mixture, which comes from the stainless-steel dust.

Most of the metallic absorbents used in cement matrix composites are steel fibers and ferrites. Yang *et al* ^16^. have studied the wave absorbing property of steel fiber reinforced concrete and found that a 31-mm-thick sample could give a peak value of 9.8 dB in the frequency range 2–18 GHz and the frequency bandwidth of 15–28 GHz for 4 dB. By using Portland cement as the base material and fly ash as the admixture, Cao and Chung^13^ have prepared the fabricated composite, which demonstrated EMI shielding property. A 4.3 mm thick sample can have a shielding effectiveness of approximately 4 dB in the frequency range 1.0–1.5 GHz. In addition, they suggest that the existence of Fe_2_O_3_ in the fly ash can enhance shielding through absorption. In general, reflection cannot eliminate or weaken radiation, and the reflected wave may interact with the incident wave, which causes disturbance to other units or devices. Only by using electromagnetic wave absorbing materials, which could transfer the electromagnetic energy to other forms, the EMI radiation could be attenuated to the furthest extent. The metal droplets and ferrite in stainless-steel dust are better choices of wave absorbing components for cement based wave absorbing materials.

**Figure 9:**
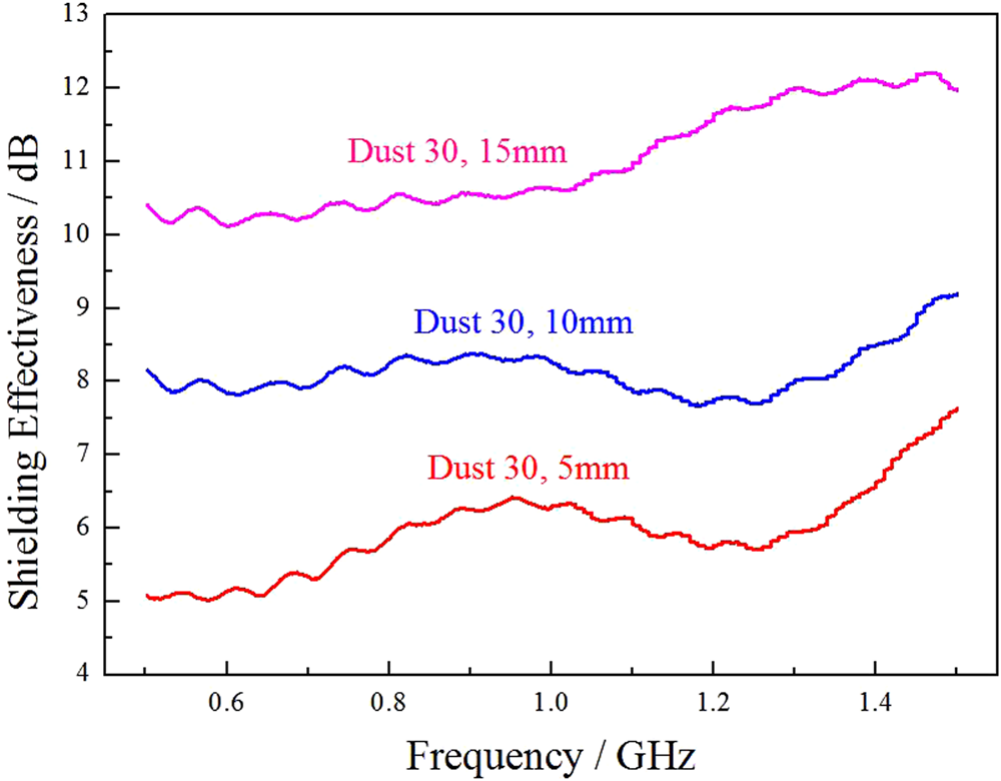
The shielding effectiveness of dust filler sample in different thickness.

The effect of sample thickness on electromagnetic shielding for the dust filler materials is shown in Fig. 9. It is clear that with the increase of sample thickness, the shielding effectiveness is enhanced. The thickness of EMI shielding material is a crucial factor to be considered in the future practical application. Stainless-steel dust, due to its low cost, has competitive advantages in some cases, for instance, large underground or remote information base construction, compared with other fillers.

There is an agreed account of efforts must be implemented to reuse the secondary resource in various methods. Stainless-steel dust is usually valorized by extracting metals or as additives for some construction materials. The present study attempts to apply it to the cement matrix for obtaining EMI shielding function, which provides a practicable and value-added way for its reutilization and/or recycling. Moreover, not only by adding the dust up directly to cement matrix, through metallurgical pre-treating, such as carbothermal reduction process, magnetization roasting, or controlled molten oxidation^17^, the EMI effect of the product prepared with that modified stainless-steel dust could be intensely improved. These emerging researches provide a new direction for the application of solid wastes in the electromagnetic field.

## Conclusions

The addition of stainless-steel dust as an admixture alone to a cement composite sample raises the EMI shielding effect. In particular, a 45 mass pct of stainless-steel dust mixture of 5 mm thickness results in the enhancement of EMI shielding effectiveness to 6–9 dB as tested in the frequency range of 500 MHz–1.5 GHz, emphasizes the incident electromagnetic wave has been reduced by approximately 60 pct. This is attributed to metal droplets and ferrite contained in the stainless-steel dust, embedded in the sample reinforced mainly the absorption of the radiation. Moreover, with the increase of sample thickness, the shielding effectiveness is also enhanced.
